# Colorimetric Hybridization Sensor for DNA Mimic of a SARS-CoV-2 RNA Marker: Direct and Inverse Bioanalysis

**DOI:** 10.1021/acsmeasuresciau.4c00043

**Published:** 2024-09-09

**Authors:** Zia ul Quasim Syed, Sathya Samaraweera, Zhuo Wang, James Kelby Schrader, Colton Scott, Joshua Schut, Dozier Johnson Smith, Joshua D. Ramsey, Sadagopan Krishnan

**Affiliations:** † Department of Chemistry, Oklahoma State University, Stillwater, Oklahoma 74078, United States; ‡ School of Chemical Engineering, Oklahoma State University, Stillwater, Oklahoma 74078, United States

**Keywords:** colorimetric sensor, SARS-CoV-2, hybridization assay, magnetic particles, RNA/DNA mimic, diagnostics

## Abstract

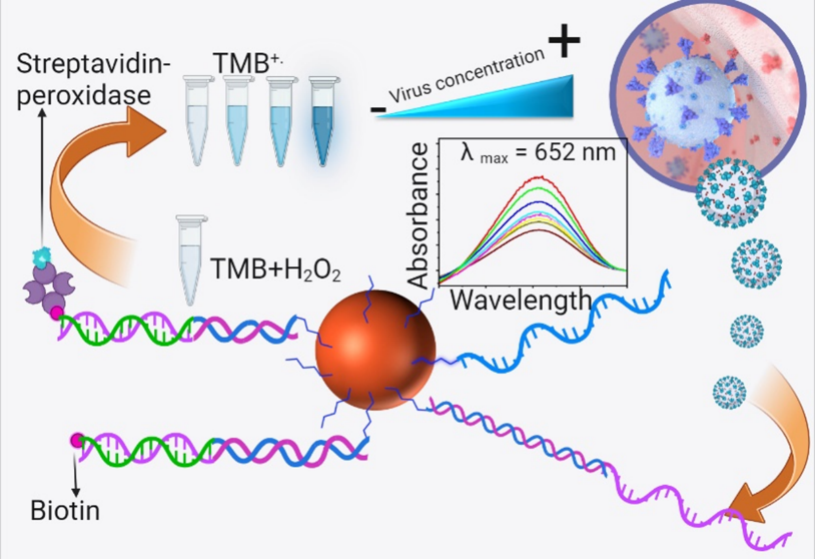

This article presents a colorimetric visual biosensor designed for direct application in undiluted biofluids, which holds significant promise for point-of-need applications. Unlike traditional biosensors that struggle with heavily diluted sample matrices, the presented biosensor does not require any instrumentation or trained personnel, making it highly practical. The sensor features an oligonucleotide probe covalently attached to magnetically separable magnetite (Fe_3_O_4_) particles. This probe selectively captures a DNA mimic of the SARS-CoV-2 RNA sequence via a base-pair hybridization. The DNA mimic oligomer sequence was tested in a buffer solution, undiluted serum, and undiluted salivary biofluids. A second complementary hybridization sequence with a biotin tag was used to bind the target oligomer already hybridized to the magnetic particle-conjugated capture probe. Subsequent detection of the target oligomer was accomplished through high-affinity selective binding of streptavidin-peroxidase labels with the detection probe biotin units for visual colorimetric detection in the presence of 3,3′,5,5′-tetramethylbenzidine and hydrogen peroxide. Inverse assaying of the unbound-free streptavidin-peroxidase labels left in the detection reagent solution offered a reverse trend to the target oligomer concentration, as anticipated. We obtained detection limits of 1 fM (buffer assay), 1 pM (undiluted serum assay), and 1 pM (undiluted saliva assay) and with the linear ranges of 1 fM–10 nM (buffer assay), 1 pM–1 nM (undiluted serum assay), and 1 pM–1 nM (undiluted saliva assay), respectively. The assays in different biofluids allowed for the estimation of the analytical performance and the effect of sample matrices on the detection limits and calibration sensitivity.

## 1 Introduction

From the 2020 pandemic, we learned that suitable alternatives to enzyme-linked immunosorbent assay (ELISA) and polymerase chain reaction (PCR) are desperately needed to rapidly and accurately diagnose contagious viruses at points of need, especially in resource-limited settings.^1−5^ However, challenges persist in sample processing, nucleic acid extraction, dilution, and amplification, thus necessitating instrument utilization and nonspecific amplification of nontarget nucleic acid materials. Instrument and clinical laboratory-based methods are unavailable in many resource-limited and remote locations.^6^ To address these limitations, continued focus on developing user-friendly, cost-effective, and instrument-free methods that can achieve ultralow detection of desired target biomolecules directly in undiluted sample matrices is critically needed.

Magnetic particles (MPs) are promising molecular carriers for diagnostic purposes because of their affordability and negligible interference with biological media.^7^ They are currently being used in the detection of bacterial and viral nucleic acids.^8^ Magnetic nanoparticles have been used for the detection of salmonella in milk^9^ and group B streptococci (GBS) DNA from the undiluted, unprocessed body fluid swab with a detection limit of 1250 CFU/mL.^10^ Magnetic particles have been used for the colorimetric detection of severe acute respiratory syndrome coronavirus-2 (SARS-CoV-2) spike protein using intrinsic peroxidase activity of magnetic particles with a sensitivity of 4.98 ng/mL^8^ and with antibody-functionalized magnetic nanoparticles using high-affinity streptavidin-coated beads with a detection limit of 84 pM.^11^ A lateral flow assay (LFA) for the SARS-CoV-2 N antibodies used Fe_3_O_4_-AgMBA@Au nanoparticles that were fabricated by embedding silver (Ag) nanoparticles with ultrathin gold (Au) shells, functionalized with 4-mercaptobenzoic acid (MBA), onto the surface of Fe_3_O_4_ through electrostatic interactions.^12^

In this study, we devised a straightforward assay with a 200 nm sized citrate-functionalized magnetic particle-based DNA sensor for its rapid separation capabilities due to higher magnetophoretic mobility^13^ (volume and concentration details are presented under the Experimental Section), negligible interference with biological media that do not require higher-temperature control. Activation of the surface carboxylic groups of the magnetic particles (25 mg/mL suspension in D.I water, 2.2 × 10^14^ particles per gram, density 1.25 g/mL sodium salt of –COOH) with a freshly made solution mixture of 1-ethyl-3-(3-dimethyl aminopropyl) carbodiimide hydrochloride (EDC, 0.4 M) and *N*-hydroxysuccinimide (NHS, 0.1 M) enabled amide bond linkages upon reacting with the amine-functionalized capture oligo solution through carbodiimide chemistry.^14^ For the capture and detection probe sequences, the readers are referred to Table S1. Free carboxyl groups that remained unreacted on the particles were blocked with 2.5 M ethanolamine to reduce nonspecific binding in the later assay steps from nontarget molecules and biocomponents present in the biofluids. The magnetically bound covalently linked capture oligomer was incubated in buffer or undiluted serum or undiluted saliva matrices to capture various concentrations of spiked SARS-CoV-2 target sequence (synthesized by IDT Technologies, Coralville, IA; Table S1). The principle of the colorimetric hybridization sensor is based on a second complementary hybridization oligomer, which has a biotin tag that allows further high-affinity interaction with streptavidin-horseradish peroxidase detection labels. This label facilitated the naked-eye colorimetric readout in the presence of added 3,3′,5,5′-tetramethylbenzidine (TMB)/peroxide (H_2_O_2_) mixture (Enhanced K-Blue, Neogen, Lexington, KY) to form a blue-colored solution with a λ_max_ at 652 nm from the oxidation of TMB to TMB^+^.^15^ The color intensity generated is proportional to the streptavidin-peroxidase-label-bound biotinylated detection probe and, in turn, the spiked target oligomer concentration. The limitation of our method is the background peroxidase activity of the magnetic particles, which can be significantly controlled by maintaining optimum conditions such as faster detection time from the enzymatic peroxidase-driven color reaction over the relatively slower particle-driven nonenzymatic activity.

## 2 Experimental Section

### 2.1 Reagents and Materials

*N*-Hydroxysuccinimide (NHS), polyethylene glycol sorbitan monolaurate (TWEEN-20), 2-aminoethanol (ethanolamine), disodium hydrogen phosphate (Na_2_HPO_4_), 2-(*N*-morpholino) ethane sulfonic acid hydrate (MES hydrate), and healthy human AB serum were purchased from Sigma-Aldrich (St. Louis, MO). Fluid MAG-CT (200 nm)-sized citrate magnetic particles were purchased from Chemicell GmbH (Berlin, Germany). Enhanced K-Blue TMB (3,3′,5,5′-tetramethylbenzidine) substrate was obtained from Neogen (Lexington, KY). Sodium chloride (NaCl) and potassium chloride (KCl) were purchased from Millipore Sigma (Burlington, MA). HRP-conjugated streptavidin, 1-ethyl-3-(3-dimethyl aminopropyl) carbodiimide hydrochloride (EDC), and potassium dihydrogen phosphate (KH_2_PO_4_) were purchased from Thermo Fisher Scientific (Waltham, MA). DNase-free water was obtained from Fisher Scientific bioreagents. Amine-functionalized capture oligomer (5′-CCA ATGTGATCTTTTGGTGT/3AmMC6T/-3′), target sequence (5′ACACCAAAAGATCACATTGGAAAAACCCGCAATCCTGCTAACAAT-3′), biotin-functionalized detection oligomer (5′-/5Biosg/ATTGTTAGCAGGATTGCGGG-3′), and storage buffer (10 mM tris, 0.1 mM EDTA, pH 8.0) were purchased from Integrated DNA Technologies, Inc. (Coralville, IA). The wash buffer composition was 10 mM PBS with 0.05% Tween-20 and 0.01% 2.50 M ethanolamine, pH 7.4. Artificial saliva made of potassium chloride, sodium carboxy methyl cellulose, potassium phosphate monobasic, potassium phosphate dibasic, magnesium chloride hexahydrate, methyl-p-hydroxybenzoate, and calcium chloride dihydrate for medical and dental research use was purchased from Pickering Laboratories (Mountain View, CA). A magnetic rack (6.73″ × 2.24″ × 2.13″) for nucleotide purification for 100–250 μL Eppendorf tubes (16 tubes) was purchased from Sergi Lab Supplies (Seattle, WA).

### 2.2 Oligonucleotide Magnetic Particle Hybridization Sensor

In each vial, 12.5 μL (0.31 mg, ∼7 × 10^10^ particles) of suspension containing carboxylated magnetic particles of diameter 200 nm (25 mg/mL, 2.2 × 10^14^ particles per gram, density 1.25 g/mL sodium salt of –COOH) was mixed with a freshly prepared solution mixture of 100 μL volume containing 0.4 M EDC and 0.1 M NHS dissolved in an MES buffer (pH 6.5) at room temperature. After a 15 min incubation period, the –COOH groups were activated into easily leaving *N*-succinimidyl ester groups, facilitating the establishment of amide bond linkages upon incubation with the amine-functionalized capture oligomer (50 μM, 200 μL, amine 3′, in 10 mM tris, 0.1 mM EDTA buffer, pH 8.0, IDT Technologies, Coralville, IA) in an ice box at 4 °C for 30 min. The resulting conjugate was subsequently separated by holding the Eppendorf tubes in the magnetic rack and removing the bulk solution, followed by two washes with 200 μL of wash buffer (0.05% Tween-20, 0.01% of 2.50 M ethanolamine in 10 mM PBS pH 7.4). To minimize nonspecific interactions of unrelated molecules upon further reaction with target oligomer spiked samples, ethanolamine (2.5 M, 100 μL, incubated for 15 min) was added to block any remaining unoccupied free surface –COOH-activated groups. The experimental identification of ethanolamine over bovine serum albumin as a more effective blocking agent in minimizing the background peroxidase-like activity of the magnetic particles is presented in the supporting document in Figure S1.

The capture oligomer-bound particles were magnetically separated and washed once with wash buffer, followed by the incubation with 100 μL of various spiked target oligomer concentrations in the range of 100 aM–10 nM (6 × 10^4^–6 × 10^12^ copies/mL) for 30 min. The sample matrices were 10 mM phosphate-buffered saline (PBS) buffer solution, undiluted commercial healthy human AB serum, undiluted artificial saliva, and undiluted unprocessed human saliva. The target sequence spiked in these matrices was captured by the magnetically bound ethanolamine-blocked capture oligomer conjugates. Following the capture step, the hybridization complex was washed twice with 200 μL of wash buffer, employing intermittent magnetic separation to remove the bulk solutions. The biotinylated detection sequence (200 μL of 50 μM, biotin at the 5′ end, in 10 mM tris and 0.1 mM EDTA buffer, pH 8.0) was then added and incubated for 30 min to facilitate the second hybridization. The complex was subsequently washed twice with 200 μL of wash buffer, and the streptavidin-HRP label (100 ng/mL, 50 μL in 10 mM PBS, pH 7.4) was incubated for high-affinity interaction of streptavidin-HRP with the biotinylated complementary probe. After an incubation period of 15 min, the unbound bulk solution from each spiked sample was collected separately in different Eppendorf tubes for measurement of the free unbound labels that would be inversely proportional to the spiked target sequence concentrations in the samples. The magnetically isolated particles from the walls of the eppendorf tubes were washed twice with the wash buffer. Finally, 25 μL of Enhanced K-Blue TMB substrate, consisting of 3,3′,5,5′-tetramethylbenzidine (TMB) and hydrogen peroxide (H_2_O_2_), was added to the particles carrying the hybridized assembly and incubated for 60 s.

The DNA mimic oligomer-hybridized magnetic particles were magnetically separated, and the supernatant was collected for spectrophotometric measurements of absorbance intensity at 652 nm (corresponding to HRP-oxidized TMB^+^).^16^ The absorbance intensity correlated with the spiked SARS-CoV-2 target sequence concentration in all biofluids, and a visual presentation of the colors was obtained by capturing pictures of the sample tubes using an iPhone. The control sample corresponds to capture oligomer-attached magnetic particles exposed to PBS buffer or biofluids. The color generation from this target oligomer unspiked control sample in the presence of an added TMB/peroxide detection reagent mixture was subtracted from the spiked sample color intensities to obtain the calibration plot. The TMB detection solution was then diluted four times for ultraviolet–visible (UV–vis) spectrophotometric measurements.

### 2.3 Instrumentation

Colorimetric absorbance measurements were performed using a CARY 100 Bio UV–visible spectrophotometer (Santa Clara, CA). Hydrodynamic diameter and zeta potentials were determined using a ZetaPALS potential analyzer (Brookhaven Instruments Corporation, Holtsville, NY). Fourier transform infrared spectroscopy (FTIR, Thermo Scientific Nicolet iS50) was operated in an attenuated total reflection mode using a diamond crystal. Characterization of the magnetic particle was done by transmission electron microscopy (TEM, JEOL JEM-2100 with Bruker EDS, the camera is model XR-40B, and imaging software is AMT Image Capture Engine V602).

## 3 Results and Discussion

### 3.1 Hydrodynamic Size and Zeta (ζ) Potential

Citrate-functionalized magnetic particles were obtained from Chemicell Inc., which displayed a zeta (ζ) potential of −53 ± 3 mV (*N* = 5) and a hydrodynamic diameter of 207 ± 3 nm (*N* = 5). After the capture sequence immobilization on the magnetic particles, the hydrodynamic size increased to 402 ± 11 nm (*N* = 5), and the zeta potential shifted more positive to −35 ± 1 mV(*N* = 5) due to the neutralization of the negative surface charges on the particles as the result of amide bond formation upon the covalent nucleotide attachment by the carboxylic acid-nucleotide amine end group coupling.

### 3.2 Shielding Intrinsic Peroxidase Mimicking Activity of Magnetic Particles

**Figure 1:**
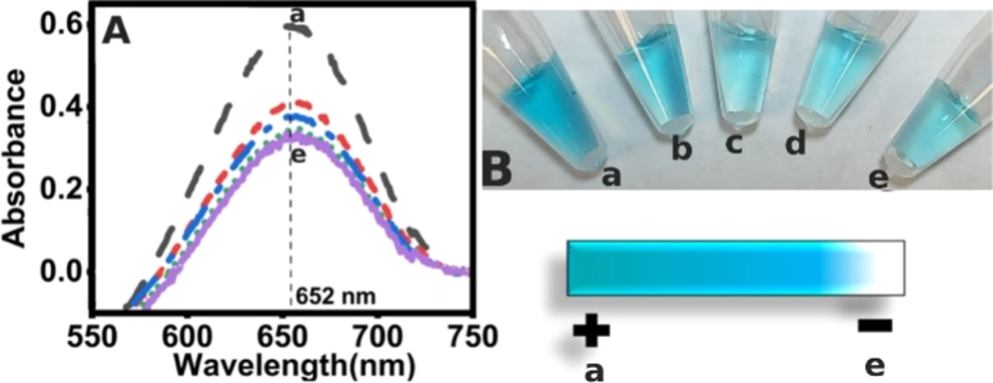
(A) UV–visible spectra upon shielding the intrinsic peroxidase mimicking activity of magnetic particles through the covalent immobilization of various concentrations of the amine-functionalized capture oligonucleotide sequence. a. No added capture oligomer; only the –COOH-activated magnetic particles in buffer and b. 12.5 μM, c. 25 μM, d. 50 μM, and e. 100 μM oligonucleotide reacted conjugates of the magnetic particles. (B). Visual presentation of the sample tubes with increased shielding and decreased background peroxidase activity of the capture probe with concentration conjugated to the magnetic particles.

The intrinsic peroxidase-like activity of magnetic particles is controllable through oligomer binding. When the oligomers are covalently linked to the magnetic particles, they form a shield around the particle, thereby preventing interaction with the TMB substrate. This is primarily attributed to physical hindrance or electrostatic repulsion.^17^ To determine the optimal condition that minimizes the peroxidase-like activity of the magnetic particles to the maximum, we reacted the EDC/NHS activated citrate-functionalized magnetic particles with various concentrations of amine-functionalized capture oligonucleotide (200 μL in 10 mM tris, 0.1 mM EDTA buffer, pH 8.0) as shown in Figure 1a–e for 0, 12.5, 25, 50, and 100 μM oligomer solutions, respectively. Followed by incubation with 25 μL of TMB/peroxide solution for 5 min, particles were separated using a magnetic rack, and the supernatant was transferred into other vials for observation and analysis. Notably, through naked-eye observation and UV–vis analysis by diluting the sample four times with 10 mM PBS, pH 7.4, we see the highest intensity for the –COOH-activated MPs (a) and the lowest absorbance in the sample with the highest concentration of capture oligonucleotide reacted (e) as presented in Figure 1A,B. Results suggested the saturation region of background signal minimization beyond 50 μM (d) as the response is nearly the same when using 100 μM (e), so we chose to use a 50 μM capture oligomer concentration in our assay for conjugating with the particles both as the optimum concentration and to preserve the expensive oligomer use. We also carried out FTIR characterization of the bioconjugate, as shown in Figure S2 and the associated text in the Supporting Information.

### 3.3 Colorimetric Oligonucleotide Hybridization Assay in 10 mM PBS

**Figure 2:**
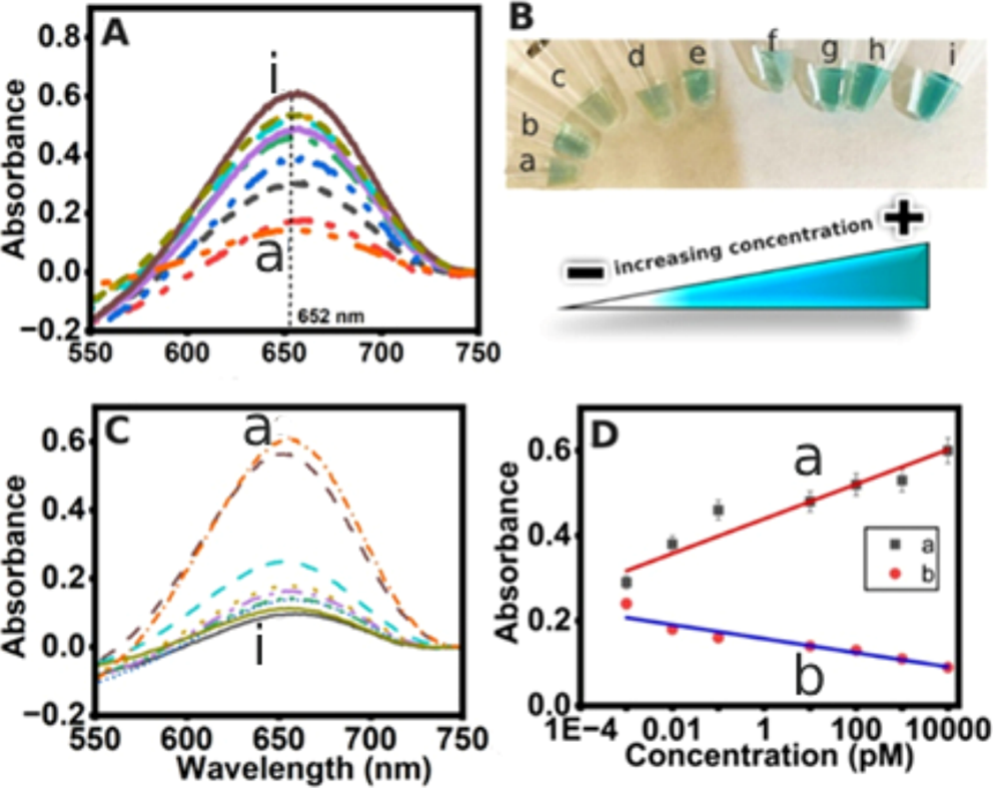
Direct (A) and inverse (C) bioanalysis. (A) Colorimetric hybridization assay response in 10 mM PBS: a. the baseline response of magnetic particle-bound capture oligomer in buffer (no spiked target oligomer). Panels b–i show the responses for 100 aM, 1, 10, 100 fM, 10, 100 pM, 1, and 10 nM SARS-CoV-2 mimic oligomer concentrations spiked in 10 mM PBS, respectively. (B) Visual presentation of the sample tubes. (C) Colorimetric signal from the unbound streptavidin-HRP labels left in the supernatant of the incubation solution that is inversely proportional to the oligomer concentration hybridized onto the particles: a. magnetically bound capture probe in buffer not exposed to any target sequence (i.e., no spiked target oligomer control). Panels b–i show the unbound or leftover streptavidin-HRP label intensity (probed by the TMB oxidation reaction) to 100 aM, 1, 10, 100 fM, 10, 100 pM, 1, and 10 nM SARS-CoV-2 mimic oligomer concentrations hybridized on the particles in 10 mM PBS, respectively. (D) Comparative plot of the absorbance of colorimetric hybridization assembly assay (a) for the magnetic particle-double hybridization assembly solution plotted against absorbance in direct proportion to the concentration of the target oligomer spiked. (b) Absorbance of the unbound streptavidin-peroxidase labels (quantitated by the TMB oxidation probe reaction) remaining in the solution after incubation with the hybridized target assembly captured with various spiked target oligomer concentrations.

We performed the assay in 10 mM PBS, pH 7.4 by spiking 100 μL of various concentrations of the SARS-CoV-2 DNA mimic oligomer (IDT Technologies Inc.) in the range of 100 aM–10 nM (6 × 10^4^–6 × 10^12^ copies/mL) and incubating them for 30 min in separate Eppendorf tubes with magnetically bound capture oligomer particles (see Section 2 for details). Figure 2A shows that with increasing target oligomer spiked concentration, the color intensity increases, and Figure 2B displays the corresponding visual picture of the sample tubes. We additionally devised an inverse assessment of successful hybridization by analyzing the unbound-free streptavidin-HRP left in the bulk solution. The underlying rationale is that with the increase in the target oligomer marker concentration hybridized on the capture oligomer-MPs, a greater extent of the second hybridization oligomer is expected to bind to the streptavidin-HRP labels, and a lower concentration of the peroxidase label is expected to remain in the bulk solution. To test this, once the magnetically separable hybridized assembly is isolated, the unbound streptavidin-HRP-labeled solution was pipetted into Eppendorf tubes and incubated with 25 μL of colorless Enhanced K-Blue solution, consisting of TMB/peroxide solution for 60 s. The intensity of the color is inversely proportional to the concentration of the bound target oligomer.

In Figure 2C, “peak a” corresponds to the magnetically bound capture probe in buffer (not exposed to any target sequence), which shows the highest absorbance because of the presence of most unbound streptavidin-HRP molecules left free in the incubation solution that was assayed for the TMB color reaction after separating out of the particles that were magnetically held onto the sample tube wall. “Peak i” corresponds to the highest concentration of target oligomer (10 nM) spiked assay, which ultimately undergoes second hybridization with the highest concentration of the detection sequence, thereby leaving behind only the lowest concentration of the streptavidin-HRP molecules in the bulk solution. This supernatant solution, when assayed based on the redox HRP labels, showed the lowest absorbance as anticipated, confirming the working principle of the designed sensor toward the target oligomer sequence.

Figure 2D(a) shows the response plot subtracted for the color intensity of the MP-capture oligomer conjugate alone in the absence of any spiked target oligomer, and Figure 2D(b) shows the assaying of unbound streptavidin-HRP labels left in solution after incubation with the hybridization assemblies on the particles. While the indirect analysis gives confidence to the analytical response, implementing only the indirect assay is not our recommendation in order to avoid any intensity decrease from unknown artifacts; hence, combining the direct and indirect analyses offers enhanced rigor to our strategy. Additionally, we performed microscopic characterization (transmission electron microscopy) to get confirmation of the immobilization of the capture oligomers on the surface of the magnetic particles, as shown in Figure S3. The average size of the air-dried functionalized magnetic particles was 14.4 ± 3.3 nm; after the immobilization of the capture oligomer on the surface of the magnetic particles, the average size increased to 50.3 ± 9.4 nm.

### 3.4 Matrix Effects

**Figure 3:**
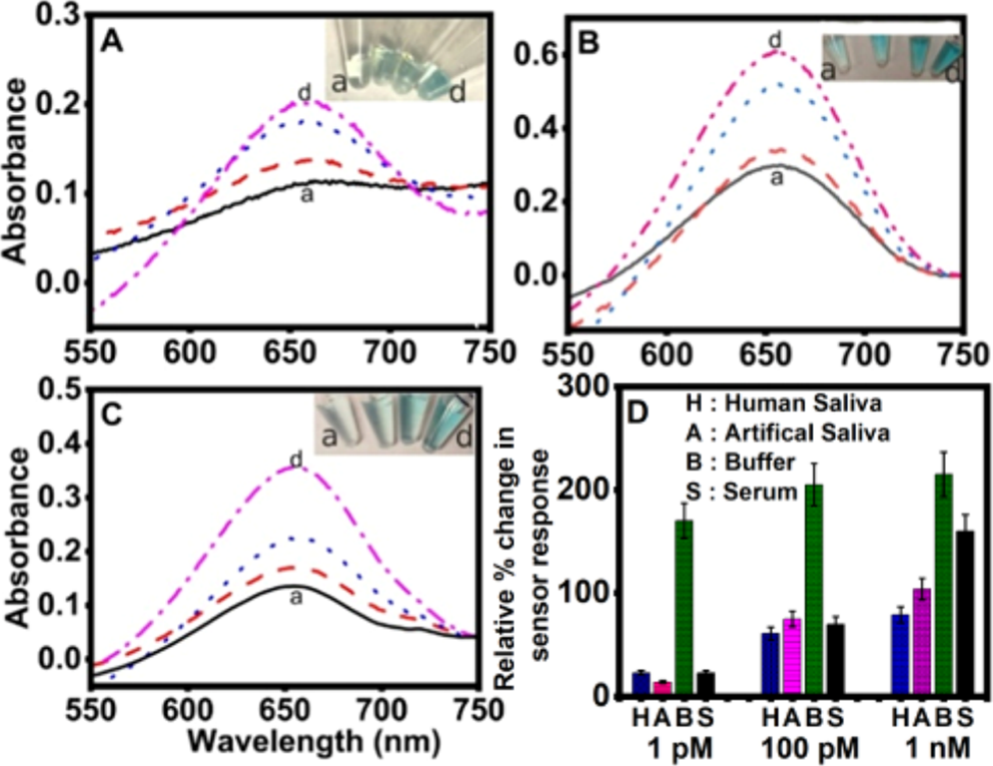
Magnetic particle-capture oligonucleotide bioconjugate-based colorimetric hybridization assay response: (A) in undiluted healthy human saliva, (B) in undiluted artificial saliva, and (C) in undiluted commercial serum. In all cases, a. control response: no spiked target oligomer; panels b–d show the responses to 1, 100 pM, and 1 nM SARS-CoV-2 mimic oligomer concentrations spiked in the respective biofluids in each assay type, respectively. (D) Bar chart comparison of the percentage absorbance changes for SARS-CoV-2 mimic oligomer spiked at three different concentrations (1, 100 pM, and 1 nM) in undiluted healthy human saliva (H), undiluted commercial artificial saliva (A), buffer (B), and undiluted commercial healthy human AB serum (S).

To check the performance of the sensor in different sample matrices, we spiked three different target oligomer sequence concentrations (1, 100 pM, and 1 nM) in undiluted healthy human saliva (Figure 3A), artificial saliva (Figure 3B), and undiluted commercial healthy human AB serum (Figure 3C). Figure 3D presents the color change comparison among the various sample matrices tested for the three representative concentrations used. Despite the compromise on the relative percentage signal changes upon advancing to biofluids from a simple buffer solution assay (Figures 3 vs 2), we are still able to detect a 1 pM concentration above the control response visually MP-capture oligomer alone in each sample matrix without any spiked target oligomer. This observation infers the usefulness of the presented approach for analysis in biofluids, and further improvement on detection limits and sensitivity is required as we focus on our ongoing research direction. According to one prior report, the viral load was 641 copies/mL in throat swab samples on day 1 of infection. For peak infection (days 5 and 6), the viral load was reported in the range of 10^4^–10^7^ copies/mL in throat swabs and sputum samples. Specifically, on day 5 (peak infection), 7.99  ×  10^4^ copies/mL for throat swab samples, 7.52 ×  10^5^ copies/mL for sputum samples, and 1.69 ×  10^5^ copies/mL for nasal swab were reported.^18^ Our sensor can detect 6.02 × 10^5^ copies/mL in 10 mM PBS (pH 7.4) and 6.02 × 10^8^ copies/mL in undiluted serum. Further modifications are required for the approach, such as improved functionalization of nanoparticles, minimization of background peroxidase activity from the magnetic particles, and improved signal amplification events to lower the limit of detection (LOD) of the designed visual colorimetric sensor to be useful for onsite and early infection detection at the point of need and on undiluted biofluids without any sample preparation or processing steps.

We attribute the lower analytical signal changes in biofluids over the simple buffer assay to the presence of other biomolecules and biological/cellular components present in biofluids, diffusion barriers to analyte hybridization with the capture oligo-bound MPs due to the highly viscous and frothy (in the case of saliva) characteristics of biofluids, nonspecific blocking of the available capture oligo sites to target oligomer, and the associated sensor fouling. The diminished analytical signal response in biofluids is greatly due to the undiluted form of the biofluids, adding more complexity than heavily diluted biofluids commonly implemented in the sensor literature, thereby effectively lowering the overall effective concentrations of unrelated biological molecules and sample viscosity.

From the comparative literature analysis presented in Table S2, we acknowledge that magnetic particles have been used for the development of various assays to detect different analytes, such as miRNAs, cancer cells, DNA, RNA of the human immunodeficiency virus (HIV), and SARS-CoV-2 spike protein, with femtomolar detection limits. However, most sensor reports involve sample dilutions, which is tedious to accomplish at the point of need. A colorimetric biosensor using γ Fe_2_O_3_ nanoparticles as peroxidase mimics was developed for the detection of the SARS-CoV-2 spike protein. The assay showed rapid color changes in real samples such as nasopharyngeal swabs, providing a practical and effective method for diagnosis.^8^

Alternatively, CRISPR/Cas 12a methodology in combination with magnetic particles has been successfully implemented for the detection of African swine fever virus, with detection limits of 20 copies/mL,^19^ and for the detection of miRNA 21.^20^ Detection of S-gene of SARS-CoV-2 with magnetic beads with CRISPR/Cas 13a has been reported. In combination with HRP and fluorescein-based antibodies immobilized on the magnetic particles, this sensor can detect 324 fM in buffer,^21^ which is much higher than our sensor. Zhong et al. used SARS-CoV-2 mimetics for highly sensitive detection in buffer with a detection limit of 84 pM, using functionalized magnetic nanoparticles (MNPs) coated with SARS-CoV-2 spike proteins for rapid detection.^11^ A recent report discussed a highly sensitive colorimetric DNA sensor platform for miRNA-155 detection using peroxidase-like activity in commercial human serum samples with an attomolar level sensitivity.^22^ However, this method has limitations: it requires five times the diluted serum samples, a temperature control of 95 °C is required for amplification of the analytical signal, and the overall assay time is approximately 4 h. Whereas our sensor is straightforward, a shorter total assay time of 2 h 20 min, it does not require any high-temperature source (Table S2).

## 4 Conclusions

Here, we demonstrated an instrument-free method for a visual colorimetric-based platform using magnetic particles to detect a DNA mimic of the SARS-CoV-2 target RNA oligomer as a model virus biomarker in buffer and undiluted biofluids. The ability to measure in undiluted clinical matrices minimizes the sample preparation steps. It provides further directions to improving the detection limits and overcoming the material limitations by devising additional signal amplification properties to the presented approach. We minimized the peroxidase-like activity of magnetic particles by optimizing the concentration of the capture oligomer and a shorter detection reaction time, wherein the peroxidase-label-based enzymatic activity toward its substrate TMB is faster than the magnetic particles.^23^ The results presented suggest that the picomolar detection of the target sequence can be achieved in undiluted matrices. The presented design methodology is not limited to a specific biomarker, and the study can be extended to test other virus markers. Similarly, the detection is not limited to serum and saliva matrices alone, and it can be further extended to other biofluids. Taken together, this research report holds significance in biomarker measurement science in various practical sample matrices.

## Supporting Information Available

Additional experimental details; materials and methods; and oligomer sequence details (Table S1); selection of blocking agent, FTIR spectra, and TEM images (Figures S1–S3); and comparative account with the representative prior literature (Table S2) (PDF)
