# Supplementary material for: Colorimetric Hybridization Sensor for DNA Mimic of a SARS-CoV-2 RNA Marker: Direct and Inverse Bioanalysis
Source: ACS Meas Sci Au. 2024 Sep 9;4(6):689–94. doi: 10.1021/acsmeasuresciau.4c00043 (PMC11659999; doi:10.1021/acsmeasuresciau.4c00043)
Supplement: Supplementary file 1 — tg4c00043_si_001.pdf [file tg4c00043_si_001.pdf]

Supporting Information

Colorimetric Hybridization Sensor for DNA Mimic of a SARS-CoV-2 RNA Marker: Direct and Inverse Bioanalysis

Zia ul Quasim Syed [a]‡, *Sathya Samaraweera* [a]‡, *Zhuo Wang* [a], *James Kelby Schrader* [a], *Colton Scott* [a], *Joshua Schut* [a], *Dozier Johnson Smith* [a], *Joshua D. Ramsey* [b], and *Sadagopan Krishnan**[a]

[a] Department of Chemistry, Oklahoma State University, Stillwater, Oklahoma 74078, United States.

[b] School of Chemical Engineering, Oklahoma State University, Stillwater, Oklahoma 74078, United States.

*gopan.krishnan@okstate.edu

Table of Contents

Preparation of buffers, oligonucleotides, and reagents S-2

Table S1. Oligomers sequence details S-3

Selection of the blocking agent (Figure S1) S-3

Spectroscopic characterization (Figure S2) S-4

Transmission electron microscopy (TEM) characterization (Figure S3) S-4

Table S2. Comparison of literature for the detection of various analytes using magnetic particles/magnetic beads S-5

References. S-6

Preparation of buffers, oligonucleotides and reagents.

Preparation of 10.00 mM phosphate-buffered saline (PBS) buffer (pH 7.4)

PBS buffer was prepared by dissolving 0.42 g of Na_2HPO_4, 0.24 g of KH_2PO_4, 8.00 g of NaCl, and 0.20 g of KCl in 100.00 mL of DNAse-free water to make a 10.00 mM PBS buffer solution. The solution's pH was adjusted to 7.4 by adding 2.00 M NaOH and 4.00 M HCl.

Preparation of 10.00 mM PBS washing buffer (pH 7.4)

0.05% Tween-20 (0.05 mL) and 0.01% of 2.50 M Ethanolamine (0.01 mL) were added to a 10.00 mL volume of 10.00 mM PBS buffer solution. After dilution to a final volume of 100.00 mL with DNAse-free water, pH correction of the resultant solution to 7.4 was attained by using 2.00 M NaOH and 4.00 M HCl.

Preparation of 25.00 mM MES Buffer (pH 6.5)

After dissolving 0.48 g of MES in 100.00 mL of DNAse-free water, 4.00 M HCl and 2.00 M NaOH were added to the mixture to adjust the pH to 6.5.

Preparation of 2.5 M Ethanolamine (pH 7.4)

An initial volume of 609.50 µL of ethanolamine (99%) was diluted with DNAse-free water to a final volume of 5.00 mL.

Preparation of EDC/NHS in MES buffer solution (4:1)

The 500 µL of MES buffer solution (pH 6.5) was used to dissolve 38.34 mg of EDC (molar mass: 191.70 g/mol) and 5.75 mg of NHS (molar mass: 115.09 g/mol).

Oligonucleotides aliquot preparation

We received 4.5 mg of amine-functionalized capture oligomer (5'- CCA ATGTGATCTTTTGGT GT/3AmMC6T/ -3'), 11.39 mg of SARS-CoV-2 target SARS-CoV-2 DNA mimic oligomer (5'-ACACCA AAAGATCACATTGGA AAA ACCCGCAATCCTGCTAACAAT -3'), and 4.10 mg biotin functionalized SARS-CoV-2 detection oligomer (5'/5Biosg/AATGTTAGCAGGATTGCGGG -3') from IDT technologies in lyophilized form in centrifuge tubes. They were reconstituted before opening as per the manufacturer's recommendation. We resuspended the oligomers in 3.44 mL, 4.15 mL, and 3.09 mL with 10 mM Tris, 0.1 mM EDTA, and pH 8.0, respectively, to obtain 200 µM stock solutions. We aliquoted the stock solutions as 100 µL vials of 200 µM solution, properly labeled the cryogenic storage tubes, and stored them at -80 ºC until each use.

Table S1. A DNA oligomer mimicking a SARS CoV-2 RNA sequence was used in this study. Amine-functionalized capture and biotinylated detection oligonucleotides are represented in bold. The starting position is referenced to the wild-type sequence. Sfold webserver[1] software was used for the statistical folding analysis of viral RNA targets.[2]

Starting target position	Ending target position	Target sequence	Antisense oligo	% GC (guanine /cytosine) content [a]	Binding Site disruption [b] (kcal/mol)[2]	Binding energy [c] (kcal/mol)[2]
28694	28713	5'-ACACCAAAAG ATCAC ATTGGA-3'	**5'-CCAATGTGATCTTTT GG T GT-$(CH_2)_3$-NH_2-3'** (capture probe covalently linked to the magnetic particles)	40	7.6	-15.8
28716	28735	5'-AAAACCCGCA ATCCTGCTAAC AAT-3'	**5' biotin-ATTGTTAGCAGGAT TGCGGG-3'** (detection probe used from solution for the second complementary hybridization with the target captured onto the particles from the first hybridization)	50	7.6	-10.4

[a] Higher the GC content, the greater the stability of the RNA[3].

[b] Lower binding site disruption energy, faster uncoiling of the RNA for hybridization[4].

[c] Lower the binding energy, higher the stability[5].

Selection of the blocking agent.

Non-specific interactions in the assay can occur between the free surfaces and non-targets, which may diminish the analytical signals and thus affect the sensitivity[6]. To minimize these interactions, blocking agents have long been used in bioassays. We performed the peroxidase-like activity test of the magnetically bound capture oligomer (50 µM, 200 µL) after blocking the free surface with 50 µL of 2.5 M ethanolamine (Figure S1 b), 50 µL of 5 % BSA (Figure S1 c), or left unblocked (Figure S1 d). We added 50 µL of Enhanced K-Blue TMB substrate, consisting of 3,3',5,5'-tetramethylbenzidine (TMB), and hydrogen peroxide (H_2O_2), and incubated for 2 min. After the incubation, we measured the absorbance of the oxidized TMB (λ_{max} at 652 nm). Curve b represents the 2.5 M ethanolamine-blocked surface that showed the lowest activity, and curve d represents the unblocked magnetically bound capture oligomer that showed the highest absorbance. Based on the results, we identified ethanolamine as being more effective than BSA. Therefore, we used 50 µL of 2.5 M ethanolamine as the blocking agent to block the free carboxyl groups on the magnetic particles. Figure S1 a represents the absorbance of D.I. water.

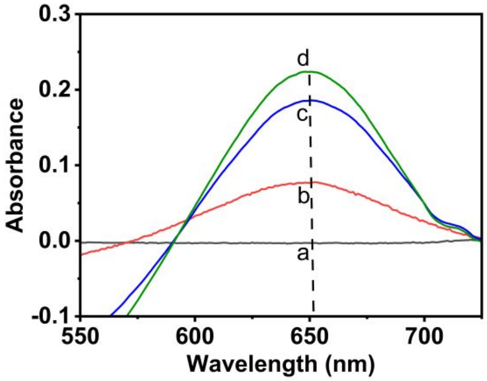

Figure S1. UV-vis spectrum of the peroxidase-like activity performed to assess the effectiveness of the blocking agent on the magnetically bound capture oligomer, **a.** represents D.I. water, **b.** UV-vis spectra of the peroxidase-like activity of the magnetically bound-capture oligomer after blocking with 2.5 M ethanolamine, **c.** UV-vis spectra of the peroxidase-like activity of the magnetically bound-capture oligomer after blocking with 5% BSA. **d.** UV-vis spectra of the peroxidase-like activity of the magnetically bound-capture oligomer without a blocking agent.

Spectroscopic characterization.

FTIR spectra results obtained showed a strong hydroxyl vibration peak at 3260 cm^{-1} from the surface carboxyl groups on the magnetic particles[7]. The vibration stretching band at 1610 cm^{-1} corresponds to the carbonyl group stretching. The additional peak at 1390 cm^{-1} was interpreted as the symmetric vibration of the ionized –COO−groups [8]. After the covalent attachment of the amine-functionalized capture oligomer with the magnetic particles to form an amide-I[9] bond, we observed a vibrational stretching band at 3290 cm^{-1}, indicating the N-H stretching vibration and confirming the covalent attachment of the capture oligomer on the magnetic particle surface. The peak at 1650 cm^{-1} corresponds to the carbonyl group in the conjugate.

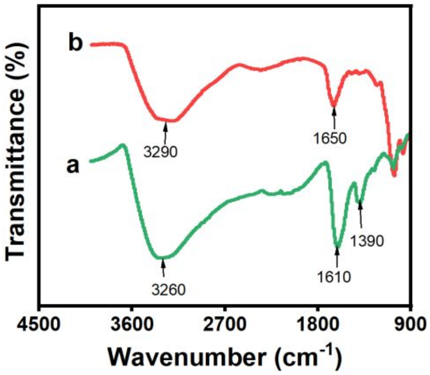

Figure S2. FTIR spectra of **a**. carboxylic functionalized magnetic particles, **b.** after the covalent immobilization of amine-functionalized capture oligomer on the magnetic particles.

Transmission electron microscopy (TEM) characterization.

We coated our magnetic particles on a carbon film-supported copper mesh grid for TEM characterization. Figure S3 presents the TEM images acquired for dried samples of magnetic particles before and after the capture oligo conjugation.

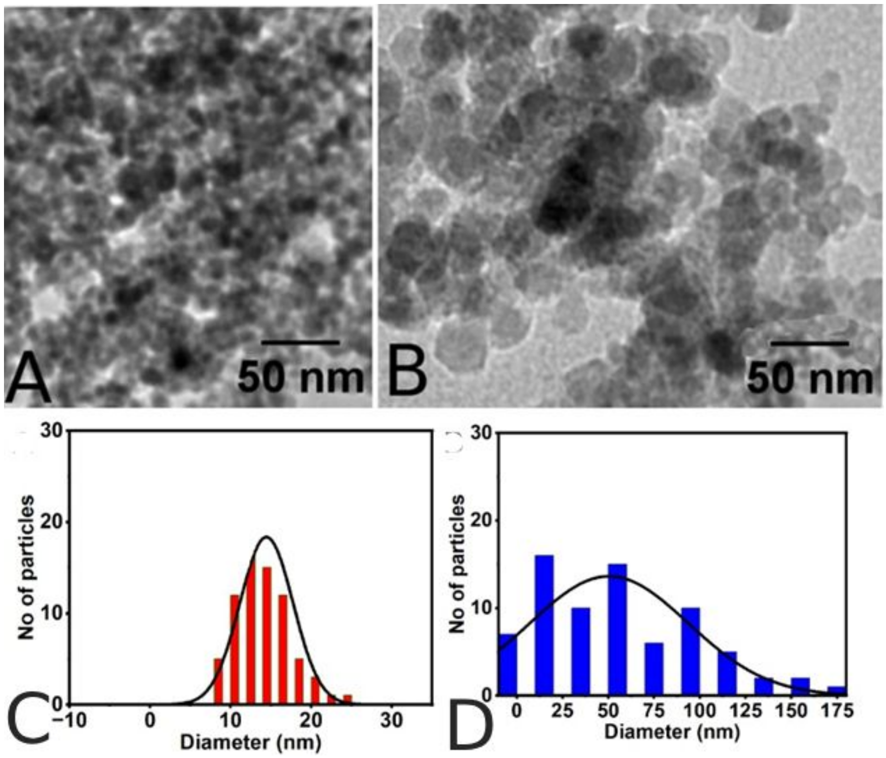

Figure S3. TEM images of **A.** magnetic particles, **B.** after covalent attachment of capture oligomer sequence to the magnetic particles, **C.** Representative size distribution histogram of magnetic particles. **D.** Representative size distribution histogram of magnetic particles-bound capture oligomer.

Table S2. Comparison of literature for the detection of various analytes using magnetic particles/magnetic beads.

Method	Analyte	Matrix	Limit of detection	Linear range	Reference
Hybridization with salt-induced aggregation with magnetic beads and gold NPs (Visual colorimetry)	Cancer cells	1:24 dilution of serum with buffer	4 cells	10 - 10^4 cells	10
Hybridization using silica-modified magnetic nanoparticles (Chemiluminescence)	HBV, HCV, HIV	1:3 dilution of serum with buffer	HBV: 16.6 pM HCV: 16.6 pM HIV: 166 pM	-	11
DNAzyme-assisted	miRNA	10 % Serum	1.5 pM	10 pM – 100 nM	12
On-particle rolling circle amplification (RCA)	miRNA	Buffer	1 fM	10 fM- 1 nM	13
DNA circuit Hybridization	miRNA-34c	PBS buffer	100 pM	50 pM – 5 nM	14

Intrinsic Peroxidase-Like Activity (Colorimetric)	Human genomic DNA	TE buffer	1.1 nM	0 – 21.5 nM	[15]
CRISPR based with streptavidin-coated magnetic particles	SARS-CoV-2	HEPES buffer	324 fM	100 fM – 1 nM	[16]
RCA-urease assisted (Visual colorimetry)	SARS-CoV-2	1:4 diluted saliva samples with buffer	0.2 pM	-	[17]
CRISPR – Cas12a (Hybridization)	miRNA-21	Clinical serum samples (dilution not mentioned)	0.5 pM	1 pM – 500 pM	[18]
CRISPR – Cas12a (Fluorescence-colorimetry)	African swine fever virus	Processed whole blood	33.2 pM	33.2pM – 33.2 µM	[19]
Single nucleotide polymorphism detection via Hybridization (Colorimetric)	Synthetic DNA	PBS buffer	0.4 fM	1 – 200 fM	[20]
Hybridization (Visual colorimetry)	DNA mimic of a SARS-CoV-2 RNA marker	PBS buffer. Undiluted serum. Undiluted human saliva. Undiluted artificial saliva	Buffer: 1 fM Serum: 1 pM Human saliva: 1 pM Artificial saliva: 1 pM	Buffer: 1 fM – 10 nM	This work

References.

(1) Ding, Y.; Chan, C. Y.; Lawrence, C. E. Sfold Web Server for Statistical Folding and Rational Design of Nucleic Acids. *Nucleic Acids Res* **2004**, *32* (Web Server), W135–W141. https://doi.org/10.1093/nar/gkh449.

(2) Moitra, P.; Alafeef, M.; Dighe, K.; Frieman, M. B.; Pan, D. Selective Naked-Eye Detection of SARS-CoV-2 Mediated by N Gene Targeted Antisense Oligonucleotide Capped Plasmonic Nanoparticles. *ACS Nano* **2020**, *14* (6), 7617–7627. https://doi.org/10.1021/acsnano.0c03822.

(3) Jacobs, E.; Mills, J. D.; Janitz, M. The Role of RNA Structure in Posttranscriptional Regulation of Gene Expression. *Journal of Genetics and Genomics* **2012**, *39* (10), 535–543. https://doi.org/10.1016/j.jgg.2012.08.002.

(4) Sasse, A.; Laverty, K. U.; Hughes, T. R.; Morris, Q. D. Motif Models for RNA-Binding Proteins. *Curr Opin Struct Biol* **2018**, *53*, 115–123. https://doi.org/10.1016/j.sbi.2018.08.001.

(5) Narlikar, G. J.; Khosla, M.; Usman, N.; Herschlag, D. Quantitating Tertiary Binding Energies of 2‘ OH Groups on the P1 Duplex of the *Tetrahymena* Ribozyme: Intrinsic Binding Energy in an RNA Enzyme. *Biochemistry* **1997**, *36* (9), 2465–2477. https://doi.org/10.1021/bi9610820.

(6) Binder, H.; Preibisch, S. Specific and Nonspecific Hybridization of Oligonucleotide Probes on Microarrays. *Biophys J* **2005**, *89* (1), 337–352. https://doi.org/10.1529/biophysj.104.055343.

(7) Wu, H.; Zhao, W.; Hu, H.; Chen, G. One-Step in Situ Ball Milling Synthesis of Polymer-Functionalized Graphene Nanocomposites. *J Mater Chem* **2011**, *21* (24), 8626. https://doi.org/10.1039/c1jm10819k.

(8) Gao, W. G.; Liu, X. C.; Chen, M. F. In Situ ATR-FTIR Investigation and Theoretical Calculation of the Interactions of Chromate and Citrate on the Surface of Haematite (α-Fe_2O_3). *RSC Adv* **2017**, *7* (65), 41011–41016. https://doi.org/10.1039/C7RA04587E.

(9) Ji, Y.; Yang, X.; Ji, Z.; Zhu, L.; Ma, N.; Chen, D.; Jia, X.; Tang, J.; Cao, Y. DFT-Calculated IR Spectrum Amide I, II, and III Band Contributions of *N* -Methylacetamide Fine Components. *ACS Omega* **2020**, *5* (15), 8572–8578. https://doi.org/10.1021/acsomega.9b04421.

(10) Yu, T.; Dai, P.-P.; Xu, J.-J.; Chen, H.-Y. Highly Sensitive Colorimetric Cancer Cell Detection Based on Dual Signal Amplification. *ACS Appl Mater Interfaces* **2016**, *8* (7), 4434–4441. https://doi.org/10.1021/acsami.5b12117.

(11) Ali, Z.; Wang, J.; Tang, Y.; Liu, B.; He, N.; Li, Z. Simultaneous Detection of Multiple Viruses Based on Chemiluminescence and Magnetic Separation. *Biomater Sci* **2017**, *5* (1), 57–66. https://doi.org/10.1039/C6BM00527F.

(12) Tian, B.; Han, Y.; Wetterskog, E.; Donolato, M.; Hansen, M. F.; Svedlindh, P.; Strömberg, M. MicroRNA Detection through DNAzyme-Mediated Disintegration of Magnetic Nanoparticle Assemblies. *ACS Sens* **2018**, *3* (9), 1884–1891. https://doi.org/10.1021/acssensors.8b00850.

(13) Tian, B.; Qiu, Z.; Ma, J.; Donolato, M.; Hansen, M. F.; Svedlindh, P.; Strömberg, M. On-Particle Rolling Circle Amplification-Based Core–Satellite Magnetic Superstructures for MicroRNA Detection. *ACS Appl Mater Interfaces* **2018**, *10* (3), 2957–2964. https://doi.org/10.1021/acsami.7b16293.

(14) Oishi, M.; Sugiyama, S. An Efficient Particle-Based DNA Circuit System: Catalytic Disassembly of DNA/PEG-Modified Gold Nanoparticle–Magnetic Bead Composites for Colorimetric Detection of MiRNA. *Small* **2016**, *12* (37), 5153–5158. https://doi.org/10.1002/smll.201601741.

(15) Öğüt, E.; Kip, Ç.; Gökçal, B.; Tuncel, A. Aggregation-Resistant Nanozyme Containing Accessible Magnetite Nanoparticles Immobilized in Monodisperse-Porous Silica Microspheres for Colorimetric Assay of Human Genomic DNA. *J Colloid Interface Sci* **2019**, *550*, 90–98. https://doi.org/10.1016/j.jcis.2019.04.089.

(16) Liu, L.; Xu, Z.; Molina Vargas, A. M.; Dollery, S. J.; Schrlau, M. G.; Cormier, D.; O’Connell, M. R.; Tobin, G. J.; Du, K. Aerosol Jet Printing-Enabled Dual-Function Electrochemical and Colorimetric Biosensor for SARS-CoV-2 Detection. *Anal Chem* **2023**, *95* (32), 11997–12005. https://doi.org/10.1021/acs.analchem.3c01724.

(17) Chang, D.; Li, J.; Liu, R.; Liu, M.; Tram, K.; Schmitt, N.; Li, Y. A Colorimetric Biosensing Platform with Aptamers, Rolling Circle Amplification and Urease-Mediated Litmus Test. *Angewandte Chemie International Edition* **2023**, *62* (51). https://doi.org/10.1002/anie.202315185.

(18) Luo, B.; Zhou, J.; Zhan, X.; Ying, B.; Lan, F.; Wu, Y. Visual and Colorimetric Detection of MicroRNA in Clinical Samples Based on Strand Displacement Amplification and Nanozyme-Mediated CRISPR-Cas12a System. *Talanta* **2024**, *277*, 126310. https://doi.org/10.1016/j.talanta.2024.126310.

(19) Mao, G.; Luo, X.; Ye, S.; Wang, X.; He, J.; Kong, J.; Dai, J.; Yin, W.; Ma, Y. Fluorescence and Colorimetric Analysis of African Swine Fever Virus Based on the RPA-Assisted CRISPR/Cas12a Strategy. *Anal Chem* **2023**, *95* (20), 8063–8069. https://doi.org/10.1021/acs.analchem.3c01033.

(20) Deng, H.; Shen, W.; Gao, Z. Colorimetric Detection of Single Nucleotide Polymorphisms in the Presence of 10-Fold Excess of a Wild-Type Gene. *Biosens Bioelectron* **2015**, *68*, 310–315. https://doi.org/10.1016/j.bios.2015.01.016.
